# A Biopsychosocial Approach to Risk and Resilience on Behavior in Children Followed from Birth to Age 12

**DOI:** 10.1007/s10578-016-0684-x

**Published:** 2016-09-15

**Authors:** Sara Agnafors, Carl Göran Svedin, Lars Oreland, Marie Bladh, Erika Comasco, Gunilla Sydsjö

**Affiliations:** 1 Faculty of Health Sciences, Division of Child and Adolescent Psychiatry, IKE, Department of Clinical and Experimental Medicine, Linköping University, 581 85 Linköping, Sweden; 2 Department of Neuroscience, Uppsala University, 751 24 Uppsala, Sweden; 3 Faculty of Health Sciences, Division of Obstetrics and Gynaecology, Department of Clinical and Experimental Medicine, Linköping University, 581 85 Linköping, Sweden

**Keywords:** Child, Genotype, Longitudinal, Mental health, Resilience

## Abstract

An increasing prevalence of mental health problems calls for more knowledge into factors associated with resilience. The present study used multiple statistical methodologies to examine a biopsychosocial model of risk and resilience on preadolescence behavior. Data from 889 children and mothers from a birth cohort were used. An adversity score was created by combining maternal symptoms of depression, psychosocial risk and children’s experiences of life events. The proposed resilience factors investigated were candidate genetic polymorphisms, child temperament, social functioning, and maternal sense of coherence. The l/l genotype of the serotonin transporter linked polymorphic region was associated with lower internalizing scores, but not mainly related to the level of adversity. An easy temperament was associated with resilience for children exposed to high adversity. Social functioning was found to be promotive independent of the risk level. The results support a multiple-level model of resilience indicating effects, though small, of both biological and psychosocial factors.

## Introduction

Increasing prevalence of mental health problems in children and adolescents raises the need for effective prevention and knowledge about how to support children at risk of dysfunctional development. Since the 1960s there has been an interest in the concept of resilience, that is, why some children at risk still develop satisfactorily [1, 2]. A commonly accepted definition of resilience, is the ability to develop well socially, mentally or physically despite exposure to stress factors commonly causing mental problems [3–5]. The view of resilience has changed from that of a static, innate capacity, to that of an acquired ability that is situational and changeable over time [1].

The link between childhood psychosocial adversities and mental health and behavioral problems is well documented [6, 7]. Childhood exposures to parental divorce, domestic violence and physical abuse have been shown to increase the risk for behavioral problems and adult depression [8–10]. Likewise, there is considerable support for an increased risk of negative behavioral and mental health outcomes as a consequence of socioeconomic deprivation [11, 12]. Moreover, maternal mental health has been shown to be associated with an increased prevalence of both internalizing and externalizing problems in children [13]. Finally, risk factors for dysfunctional development often co-occur [14], and cumulative life adversities exert the most important risk for negative outcomes [15].

Previous research on resilience has focused on many of the above mentioned adversities and exposures, and provided a number of factors associated with a better-than-expected development in children growing up in a detrimental environment. These resource factors can be grouped into three domains: internal characteristics, family environment, and social environment. *Internal characteristics* represent the child’s inner strengths and capacities such as temperament, learning ability, self-esteem and adaptive skills. Temperament has been shown to specifically affect behavior in school-aged children [16] and to be associated with resilient outcomes in adults [17]. *Family factors* found to be associated with resilience are attachment, parenting styles and the parent–child relationship, all impacting the parent–child interaction. Maternal sense of coherence (SOC) has been found to be associated with lower levels of behavioral problems in preschool children [18]. Likewise, an association between maternal SOC and child attachment style and socioemotional adjustment has been found [19]. Resource factors found in the *social environment* are school performance, pro-social adult relations and good friends. These resource factors have shown a convincing replicability over the years [14].

The research on resilience has evolved from what is called the first wave, describing correlates of resilience, through the second and third wave which aim to explain the processes behind resilience correlates and implementing research into intervention programs. Now there is a fourth wave emerging which views resilience as a multilevel (bio-psycho-social) construct [20]. As the fourth wave of resilience research arises, there is a call for a multiple-level-of analysis, that is, the incorporation of biological measures in the prevailing psychosocial-environmental perspective [21]. During the last decades, an increasing interest to include a biological approach in human resilience research has been evident [22–26]. Caspi et al. (2002) showed that a genotype related to low monoamine oxidase (*MAOA*-uVNTR) activity was associated with antisocial behavior in the presence of childhood maltreatment; consequently a high MAOA activity had a protective effect in individuals exposed to childhood maltreatment [27]. Following the lead of Caspi and colleagues with the subsequent finding of an interaction effect of a functional polymorphism in the serotonin transporter gene (5-HTTLPR) and stressful life events on depression [28], gene-by-environment interaction studies have flourished. Results have been inconsistent and meta-analyses including the most studied genotype (5-HTTLPR) have come to different conclusions [29, 30]. However, there is support for gene-by-environment interactions including not only the 5-HTTLPR on depression and the MAOA-uVNTR on antisocial behavior [31], but also linking two other functional polymorphisms *BDNF* Val66Met and *COMT* Val158Met respectively in combination with stressful life events to depression [32]. Kaufman et al. (2006) found a four-way interaction between maltreatment history, *BDNF* genotype, *5*-HTTLPR genotype, and social supports, where social supports decreased depression scores for maltreated children who were carriers of the *5*-HTTLPR s/s genotype and the *BDNF* Met-allele [32]. Another study found an interaction effect between the *COMT* Val158Met genotype and recent stressful life events on depression onset [33]. Despite contradictory results, these four genotypes have thus been well studied in relation to mental health. Considering the lack of an overlap between the established resilience research and the growing body of gene-by-environment research, there is a request for new research strategies combining these areas of knowledge [4, 34].

One key feature of the study of resilience is that it has to be carried out in a longitudinal perspective [35]. The South East Sweden Birth Cohort study (SESBiC-study) offers the possibility to follow a birth cohort from the age of 3 months to the age of 12. The SESBiC-study started in 1995 with the purpose of early identification of psychosocially burdened families where children were at risk of dysfunctional development [36–38]. Information on maternal and child psychosocial and medical health has been obtained at baseline and the 3 and 12-year follow-ups, including numerous factors of risk and resilience.

Combining different statistical methods in resilience research can better evaluate the consistency of the results [39]. As previously done by Miller-Lewis et al. [40] this study builds upon multiple methodologies using both variable-centered and person-centered approaches. The present study adds to the existing literature by: (1) investigating both biological and psychosocial factors of resilience, (2) examining risk- and resilience factors in early childhood and their long-term impact on emotional and behavioral problems measured in preadolescence, and (3) using multiple methodologies to evaluate the strength of the results.

### Aim

**Fig. 1:**
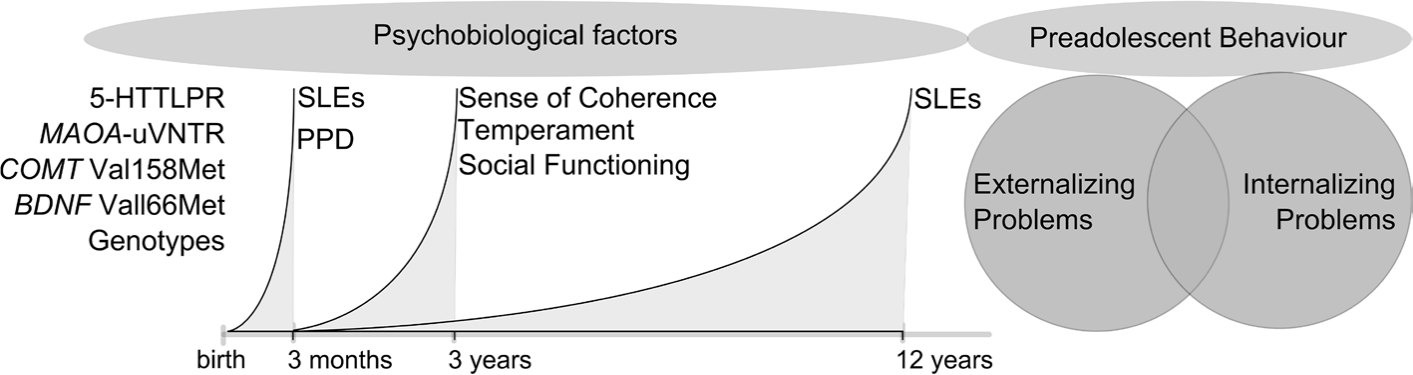
Conceptual model of the study design. Promotive factors are hypothesized to impact the risk for behavioral problems in preadolescence in children exposed to early life adversity. *5*-HTTLPR serotonin transporter gene-linked polymorphic region, *MAOA* monoamine oxidase A, *COMT* catechol-o-methyl transferase, *BDNF* brain derived neurotrophic factor, *SLE* stressful life events, *PPD* postpartum depression

The aim of this study was to examine proposed resilience factors at pre-school age and their impact on child behavior at the age of 12, using a biopsychosocial model of risk and resilience. More specifically, maternal factors such as sense of coherence as well as individual factors such as temperament, social functioning and genotype were hypothesized to have a protective effect on child behavior in children exposed to cumulative adversities. The intention was also to examine whether the proposed salutogenic factors were specific for children at risk of dysfunctional development (resilience factors), or if they were generally promotive for all children (resource factors), using both variable-centered and person-centered methods. Figure 1 illustrates the conceptual model of the study.

## Subjects and Methods

### Subjects

All mothers of the children from a geographical birth cohort born between May 1st 1995 and December 31st 1996 in southern Sweden were asked to take part in the study, whereof 1723 mothers (88 %) agreed to participate. The mean age of the mothers was 28.2 ± 4.6 years at child birth. Ninety-six percent of the mothers (*n* = 1574) were cohabitating, 3.5 % (*n* = 57) were single parents, and 0.5 % (*n* = 8) reported other family arrangements. Most mothers were born in Sweden (88.6 %, *n* = 1482), but 6.2 % (*n* = 103) were born in Europe, and 5.3 % (*n* = 88) outside Europe. Of the newborn children, 52.8 % were boys and there were 27 twin pairs.

At the 3-year follow-up, one child was deceased and 1452 (84 %) agreed to participate. At the 12-year follow-up, two children and four mothers were deceased. Ten children had moved out of the country and 24 had intellectual disabilities and could therefore not participate, which left 1683 eligible participants of whom 889 (52.8 %) chose to take part.

### Procedure

The baseline study was carried out at Child Welfare Centers, (CWC) in connection with the routine 3-month check-up. Information about the study was given by the CWC staff. Standardized instruments were administered, and the mothers were also interviewed by a psychologist in order to gain more information about the families’ psychosocial status. The 3-year follow-up was done in connection with the routine examination of 3-year olds at the CWC. Mothers were asked to fill in questionnaires and medical information was retrieved from the child’s medical records. At the 12-year follow-up, information letters were sent to eligible families, i.e. custodial parents, by mail. A separate simplified information letter was enclosed for the child. The follow-up was carried out at school where research assistants met with the children in small groups. The children provided saliva samples for genetic analyses and answered a questionnaire as part of a larger study. Children who no longer lived in the area or were not in school that day were scheduled for a home visit or a meeting at their new school. The mothers were sent a package of questionnaires by mail. Participating families received two movie tickets.

### Instruments

#### Baseline

The Life Stress Score (LSS) is a 50-item semi-structured interview form consisting of three main domains regarding social, medical and psychological conditions. The social domain covers 17 items regarding the mother’s social situation, that is family structure/social network, education, occupation and living conditions, whereas the medical domain holds 17 items which focus on medical information, i.e. personal maturity, health, workload, pregnancy and health care utilization. Psychological information is obtained through 16 questions about traumatic experience during childhood and adulthood respectively, pregnancy and child birth and relationship with the child. The LSS has been used previously in a Swedish population-based study [41], and was filled out by a psychologist after interviewing the mothers at baseline.

The Edinburgh Postnatal Depression Scale (EPDS) [42] is a widely used ten item self-report questionnaire designed to screen for postnatal depression. Every item is ranged 0–3, with a total score of 30. The EPDS is not by itself diagnostic, but with a cutoff level of 9/10 the sensitivity of 96 % for Major Depression and the specificity of 62 % has been noted [43]. EPDS refers to the 7 days preceding completion of the form and was filled out by the mothers at baseline.

#### 3-Year Follow-Up

At the 3-year follow-up all assessments were completed by the mothers. The Coddington life event scale (CLES) [44] is a form used to screen for exposure to different life events, for example parental divorce, domestic violence and serious illness or injury within the family. A modified version of the original scale was used, consisting of virtually the same events as the CLES, however, it only evaluates the occurrence of an event, but not when it occurred. It consists of 32 specific items and one open-ended item which can include any events not stated in the list. The mothers were instructed to report life events within the family since the birth of the participating child. The CLES is a widely used instrument, and similar versions have been used in Swedish population-based studies [45].

The Sense of Coherence form (SOC) [46] is a widely used form measuring factors associated with good coping ability. As part of Antonovskys’ concept of health, it focuses on the three main domains of comprehensibility, manageability and meaningfulness. In this study, the Swedish version of the 13 item SOC was used. Every item is graded on a seven point scale, where 1 corresponds to low agreement and seven to high agreement. The 13 items are compiled into a total score, resulting in the single global factor SOC. The SOC form has been shown to exhibit good validity [47].

Child temperament was assessed by a global construct “difficult child”, based on concepts from studies by Thomas and Chess [48]. The form includes 11 questions regarding adaptability to altered situations, intensity of emotional reactions and quality of mood. The first nine questions are scored on a five-point Likert scale assessing the child’s temperament compared to other children. The last two questions are ranged 1–3 and 1–7 respectively, and were consequently adapted to give equal weight as the other items. The scores were then reversed so that a high score indicated an easy temperament.

As a measure of social functioning, 13 items from the Child Behavior Check List (CBCL) 2/3 were used. As the 2/3 year version does not contain a social problems subscale, these 13 items were specifically chosen to reflect social functioning and thus included items concerning communication, cooperative capability and shyness/parental dependence. The items were scored on a three-point Likert scale ranging from “not true” to “very true or often true”. The result of the summed score was then reversed, so that a high score indicated good social functioning.

#### 12-Year Follow-Up

The Child Behavior Check List/4–18 (CBCL) is a 113 item form assessing child behavior [49]. There are eight subscales which also form the broadband symptom scales of internalizing and externalizing problems. The CBCL is extensively used, and has been shown to exhibit good validity and reliability [50]. The CBCL/4–18 was answered by the mothers at the 12-year follow-up.

Potentially traumatic life events experienced by the child, was assessed by the Swedish version of Life Incidence of Traumatic Experience (LITE) [51]. The form holds 16 items; 15 describing traumatic events and one open item for any upsetting or scaring event not described in the checklist. The LITE was filled out by the children at the 12 year follow-up. Results on the LITE form were dichotomized into <90th percentile and ≥90th percentile.

### Genetic Analysis

The non-invasive and all-in-one Oragene® DNA Collection Kit (DNA Genotek) was used for the collection, stabilization and transportation of saliva samples. DNA was isolated according to the laboratory protocol for manual purification of DNA. *BDNF* Val66Met A/G SNP (rs6265), COMT Val158Met SNP (rs4680), MAOA-uVNTR and *5*-HTTLPR genotyping was carried out according to standardized protocols.

The genotyping call was blind to psychosocial data. In order to estimate the quality-rate of genotyping errors, a random repetition of ~13 % of the sample was carried out; the comparison indicated no inconsistencies. The genotypes were in Hardy–Weinberg equilibrium: *5*-HTTLPR *p* = 0.10; females *p* = 0.51; males *p* = 0.10, *BDNF* Val66Met *p* = 0.77; females *p* = 0.27; males *p* = 0.16, COMT *p* = 0.87; females *p* = 0.21; males *p* = 0.320, MAOA-uVNTR females *p* = 0.83 respectively.

### Data Analysis

Dichotomous variables were created for the protective variants and risk variants of each genotype, respectively: COMT Val158Met, genotypes homozygous for the Val allele versus carriers of the Met allele; MAOA uVNTR, carriers of high activity allele (34R and 44R) versus homozygous of the low activity allele (3R); *5*-HTTLPR, genotypes homozygous for the long allele versus carriers of the short allele and *BDNF* Val66Met, genotypes homozygous for the Val allele versus carriers of the Met allele.

A cumulative early life adversity index was calculated by combining the LSS, EPDS and CLES. Bivariate linear regression indicated somewhat similar effect sizes for the LSS, EPDS and CLES on internalizing and externalizing problems (β = 0.196–0.436), where all 95 % confidence intervals overlapped (i.e. no statistically significant differences between the coefficients were present), therefore they were all given equal weight. Multicollinearity was also tested for, and as the multicollinearity was found to be weak to moderate (0.15–0.30) though not expected to have significant effect on the estimates, no further adjustments were made. The scores on each instrument were standardized to allow summering.

Different statistical methodologies were used to examine resilience to behavioral problems at age 12, as previously done by Miller Lewis et al. [40]. The idea is to evaluate data from a variable-centered approach (hierarchical regression and residual regression) as well as a person-centered approach. In the first approach ordinary statistical models are applied to the data while in the person-centered approach groups that are similar within but different between are created prior to any statistical analysis. These two approaches complement one another and allow a more substantial analysis of the data. All three methodologies examined internalizing and externalizing problems separately. Initially, bivariate analyses were conducted in order to examine both proposed risk- and resilience factors. Only factors that were significantly associated with internalizing or externalizing problems at age 12 were included in further analysis. This was done in order to avoid saturated models, especially with the interaction models.

The first model consisted of hierarchical multiple linear regressions, including statistical interactions between proposed risk and resilience variables. In this model, significant interaction effects would possibly indicate a protective effect in the context of adversity. At step one, the covariates ethnicity, sex and experience of traumatic life events by age 12 were entered. At step two, cumulative risk was entered and at step three proposed resilience factors were added. At the final step, interaction terms between cumulative risk and all proposed resilience factors were entered.

The second model utilized linear regression to compute resilience residual variables. This was done by regressing the level of behavioral problems on the risk score (cumulative early life adversity). The residual scores were reverse-coded whereby higher scores indicated greater resilience with respect to behavioral problems [40]. Consequently, children who scored above the fitted regression line, that is, a “better-than-expected” outcome, were considered resilient, whereas children who scored below the fitted regression line (a “worse-than-expected” outcome), were assumed to be more vulnerable. The residuals were subsequently used as outcome variables in a following regression, with proposed resilience factors entered as predictor variables. Multivariate analyses were then run separately for the lowest and highest third of cumulative risk scores, allowing for comparison of effect sizes between children facing low and high adversity.

The third model used a person-centered approach. Risk scores and behavioral problem scores were divided into thirds, enabling the creation of four groups as used initially by Masten et al. 1999 [39]: the *resilient* group with a top third adversity score and a lowest third behavioral problem score; the *maladaptive* group with a top third score of both adversity and behavioral problems; the *competent* group scoring in the lowest third on both adversity and behavioral problems and the *highly vulnerable* group with a score in the lowest third on adversity but in the highest third of behavioral problems. The categorization was done for internalizing and externalizing problems respectively. The middle third on either variable were not included in further analysis. Next, comparison of groups was performed to examine whether proposed resilience factors had different effects in children of low versus high adversity with high versus low functioning.

Results are presented with corresponding regression coefficients β and 95 % Confidence Intervals (CI). A *p* value <0.05 was considered statistically significant. Statistical analyses were performed using IBM SPSS version 20 (IBM Corporation, Armonk, NY).

### Drop-Out Rate Analysis

The total drop-out rate was 47.2 % (*n* = 794). There was a difference when comparing immigrant status between participants and non-participants at the 12-year follow-up, where 54.6 % (*n* = 802) of mothers born in Sweden (*n* = 1468) took part compared to 44.6 % (*n* = 45) of mothers born in Europe (*n* = 101) and 34.9 % (*n* = 30) of mothers born outside of Europe (*n* = 86) (*χ* ^*2*^ = 15.792, *p* < 0.001). Likewise, differences were found between participants (*m* = 13.56, *SD* = 6.35) and non-participants (*m* = 14.56, *SD* = 7.59) at the follow-up when a comparison for experience of cumulative life adversities was made (*t* = 2.908, *p* = 0.004).

### Ethical Approval

The study outline was approved by the Ethics committee at the University of Lund in 1994 and 1998 and by The Regional Ethical Review Board in Linköping in 2007.

## Results

### Bivariate Analysis

Proposed risk and resilience factors were first tested in bivariate linear regression to examine whether they were significantly associated with internalizing and externalizing problems. Genetic polymorphism were run both separately and in interaction with cumulative life adversities as the hypothesis was to find gene-by-environment effects rather than main effects. Cumulative life adversity increased the risk for both internalizing and externalizing problems (β 0.16, CI 0.11–0.21; β 0.21, CI 0.15–0.26). A high maternal sense of coherence was significantly associated with a lower degree of behavioral problems (β −0.10, CI −0.13 to −0.07; β −0.11, CI −0.15 to −0.08). Likewise, an easy temperament (β −0.19, CI −0.25 to −0.13; β −0.26, CI −0.33 to −0.19) and good social functioning (β −0.53, CI −0.66 to −0.39; β −0.60, CI −0.76 to −0.44) was also associated with a decreased risk for internalizing and externalizing problems. Carriers of the l/l genotype of the *5-*HTTLPR had less internalizing problems compared to s-carriers (β −1.01, CI −1.70 to −0.32), but no other main genetic or gene-by-environment effects were seen. All analyses including the genetic polymorphisms were also run separately for females and males, but no significant effects were found. Girls had an increased risk for internalizing problems compared to boys (β 0.71, CI 0.09–1.32), and boys had a higher risk for externalizing problems (β −1.24, CI −1.95 to −0.52). Children whose parents were both born in Sweden had a lower risk of externalizing problems compared to second generation immigrants (β 1.32, CI 0.18–2.47). Experience of traumatic life events by age 12 increased the risk for externalizing problems (β 2.33, CI 1.00–3.67), but not for internalizing problems (β −0.52, CI −1.68 to 0.65).

### Model I Interaction

Interaction terms were entered in step four, which was the final model. Effect sizes for all interactions were very small and thus not likely to be of clinical significance. Cumulative life adversities did not stay significant in the final model. Good social functioning in children was associated with a lower risk for both internalizing and externalizing problems (Table 1). The association also remained for maternal sense of coherence, although effect sizes stayed weak. The l/l genotype of the *5*-HTTLPR predicted lower internalizing and externalizing scores while an easy temperament predicted lower externalizing scores. No effect was seen for ethnicity in the final model. Experience of traumatic life events by age 12 increased the risk for externalizing problems but not for internalizing problems. An interaction effect between child temperament and cumulative life adversities was seen for both internalizing and externalizing problems (Table 1).

**Table 1:** Model I: multiple regression predicting internalizing and externalizing problems

Variables	Internalizing problems			Externalizing problems		
	β	CI	*p*	β	CI	*p*
Step 1						
Sex	0.93	0.26–1.61	0.007	−0.93	−1.70 to −0.16	0.018
Ethnicity	0.89	−0.22–2.00	0.116	1.65	0.38–2.93	0.011
LITE	−0.26	−1.54–1.02	0.688	2.00	0.54–3.47	0.007
Step 2						
Sex	1.05	0.40–1.71	0.002	−0.78	−1.53 to −0.04	0.040
Ethnicity	0.22	−0.88–1.32	0.693	0.83	−0.43–2.08	0.197
LITE	−0.59	−1.83-0.66	0.356	1.602	0.18–3.03	0.027
CELA	0.60	0.42–0.77	<0.001	0.74	0.54–0.94	<0.001
Step 3						
Sex	0.90	0.26–1.53	0.006	−0.95	−1.68 to −0.23	0.010
Ethnicity	−0.29	−1.37-0.80	0.602	0.33	−0.90–1.57	0.595
LITE	−0.54	1.74−0.67	0.382	1.71	0.34–3.07	0.015
CELA	0.35	0.16–0.54	<0.001	0.44	0.22–0.66	<0.001
SOC (m)	−0.05	−0.08 to −0.01	0.005	−0.05	−0.09 to −0.01	0.013
Social functioning (c)	−0.33	−0.49 to −0.18	<0.001	−0.34	−0.52 to −0.16	<0.001
Temperament (c)	−0.07	−0.13–0.00	0.053	−0.14	−0.21 to −0.07	<0.001
*5*-HTTLPR (c)	−0.97	−1.66 to −0.30	0.005	−0.84	−1.61 to −0.07	0.033
Step 4						
Sex	0.89	0.26–1.52	0.006	−0.98	−1.69 to −0.26	0.007
Ethnicity	−0.07	−1.15–1.01	0.902	0.53	−0.70–1.75	0.401
LITE	0.64	−0.55–1.833	0.293	1.60	0.25–2.95	0.021
CELA	−0.30	−1.50–0.91	0.632	0.63	−0.75–2.00	0.371
SOC (m)	−0.05	−0.09 to −0.02	0.002	−0.05	−0.09 to −0.02	0.006
Social functioning (c)	−0.37	−0.53 to −0.21	<0.001	−0.36	−0.54 to −0.17	<0.001
Temperament (c)	−0.06	−0.13–0.01	0.072	−0.14	−0.22 to −0.07	<0.001
*5*-HTTLPR (c)	−0.93	−1.61 to −0.25	0.007	−0.81	−1.64 to −0.09	0.041
CELA × SOC	0.01	−0.01 to 0.03	0.072	0.01	0.01–0.03	0.175
CELA × social functioning	0.03	−0.03–0.09	0.307	0.03	−0.04 to 1.00	0.465
CELA × temperament	−0.04	−0.07–0.00	0.035	−0.06	−0.10 to −0.03	0.001
CELA × *5*-HTTLPR	−0.08	−0.43–0.27	0.644	0.07	−0.47 to 0.33	0.738

Multiple regression predicting child internalizing and externalizing problems at age 12, with proposed resource variables measured at the child’s age 3

Dependent variables: CBCL (internalizing and externalizing symptoms). Independent variables: cumulative early life adversity, maternal sense of coherence, child social functioning, child temperament *5-*HTTLPR (0 = l/l, 1 = s-carriers), sex (1 = boys and 2 = girls) and ethnicity (0 = both parents born in Sweden, 1 = one or both parents born abroad), LITE (0 < 90th percentile, 1 ≥ 90th percentile)

*SOC* sense of coherence, *5*-HTTLPR serotonin transporter gene-linked polymorphic region, *CELA* cumulative early life adversity, *LITE* life incidence of traumatic events, *m* maternal variable, *c* child variable

### Model II Residuals

Resilience residual variables were created by regressing the level of behavioral problems on the risk score (cumulative early life adversity). The residuals were thereafter used as outcome variables in a following regression, with proposed resilience variables entered as predictor variables. Multivariate analyses were then run separately for the lowest and highest third of cumulative risk scores. For internalizing problems 553 children (62.2 %) had better outcomes than expected, meaning a positive residual. For externalizing problems 560 children (63.0 %) had a better-than-expected outcome. Hierarchical multiple regression was performed with residual scores for internalizing and externalizing problems respectively. At step one, sex and ethnicity were entered, and at step two proposed resilience factors were added. The analyses were run first for the whole sample, and then for low and high adversity groups separately.

It may appear that the effect sizes are small; however, given that the residual scores are standardized and therefore normally range between −2 and +2, the coefficients are of expected sizes (Table 2). Regarding internalizing problems, female sex was significantly associated with a higher residual score both in the whole group and in the high risk group. Maternal sense of coherence had a small positive effect in the whole sample. Good social functioning in children was associated with lower residual scores for both the total, low and high adversity groups, while an easy temperament implied a small positive effect in the whole sample and in the high risk group. The l/l genotype of the *5-*HTTLPR was significantly associated with lower residual scores in the total sample and in the low adversity group.

**Table 2:** Model II: multiple regression predicting internalizing and externalizing standardized resilience residuals for the whole sample and for low and high risk groups separately

Variables	Internalizing problems			Externalizing problems		
	Whole sample β (n = 889)	Low CELA β (n = 246)	High CELA β (n = 238)	Whole sample β (n = 889)	Low CELA β (n = 246)	High CELA β (n = 238)
Sex	0.18*	0.11	0.42*	−0.20*	−0.15*	−0.20
Ethnicity	−0.09	−0.46	−0.29	0.03	0.05	−0.24
LITE	0.13	0.01	0.26	0.31*	0.02	0.22
SOC (m)	−0.01*	−0.01	−0.01	−0.01*	−0.01	−0.01
Social functioning (c)	−0.08**	−0.09*	−0.08*	−0.07**	−0.05*	−0.08*
Temperament (c)	−0.02*	−0.01	−0.04*	−0.03**	−0.01	−0.05*
*5*-HTTLPR (c)	−0.22*	−0.23*	−0.16	−0.17*	−0.14	−0.21

Linear regression models predicting child internalizing and externalizing problems at age 12, with proposed resource variables measured when the child was age 3

Dependent variables: CBCL (internalizing and externalizing symptoms). Independent variables: maternal sense of coherence, child social functioning, child temperament. Categorical variables: *5*-HTTLPR (0 = l/l, 1 = s-carriers), sex (1 = boys and 2 = girls) and ethnicity (0 = both parents born in Sweden, 1 = one or both parents born abroad), LITE (0 < 90th percentile, 1 ≥ 90th percentile)

*CELA* cumulative early life adversity, *LITE* life incidence of traumatic events, *SOC* sense of coherence, *5*-HTTLPR serotonin transporter gene-linked polymorphic region, *m* maternal variable, *c* child variable

*p < 0.05

**p < 0.001

Regarding externalizing problems, male sex was significantly associated with a higher residual score in the whole group as well as in the low risk group. Experience of traumatic life events increased the risk for externalizing problems in the total group. A high maternal sense of coherence reduced the residual score in the whole sample (Table 2). Good social functioning in children had a promotive effect on externalizing resilience residuals in all three groups, while an easy temperament had a promotive effect in the total sample and in the high adversity group. The l/l genotype of the *5-*HTTLPR was significantly associated with lower residual scores in the total sample but not when the groups were divided by the level of risk.

### Model III Person-Centered Approach

Four groups were created by combining the lowest versus highest tertiles of risk scores and behavioral problems for internalizing and externalizing problems respectively. For distribution, see Tables 3 and 4. Chi square tests showed that groups differed with respect to behavioral problems regarding both internalizing (*χ* ^*2*^ = 474.01, *p* < 0.001) and externalizing problems (*χ* ^*2*^ = 445.18, *p* < 0.001). This result is in line with the assumption that a high level of adversity is associated with a high degree of behavioral problems, and that resilience is to be considered a “better-than-expected” outcome [40].

**Table 3:** Model III: MANCOVA results—resource variable means or medians for the four adaption groups and planned contrast for internalizing problems

Resource variable	A. Resilient (high CELA + low int probl) n = 106 M (SD)	B. Maladaptive (high CELA + high int probl) n = 124 M (SD)	C. Competent (low CELA + low int probl) n = 159 M (SD)	D. Highly vuln (low CELA + high int probl) n = 87 M (SD)	Planned contrast a/b, a/c, a/d, b/c, b/d, c/d
SOC (m)	64.93 (11.260)	62.79 (10.86)	73.38 (8.47)	70.75 (9.17)	a/c <0.001, a/d 0.001, b/c <0.001, b/d <0.001, c/d 0.001
Social functioning (c)	17.35 (1.998)	15.72 (2.97)	17.87 (1.66)	16.74 (2.27)	a/b <0.001, b/c <0.001, b/d 0.012, c/d <0.001
Temperament (c)	22.81 (5.000)	20.06 (5.25)	24.82 (5.13)	22.64 (4.68)	a/b <0.001, a/c 0.006, b/c <0.001, b/d 0.001, c/d 0.004
*5*-HTTLPR (c), *M* (range)	1 (1)	1 (1)	1 (1)	1 (1)	a/d 0.011,c/d 0.018
Sex, *M* (range)	1 (1)	1 (1)	1 (1)	1 (1)	a/b 0.014, a/c 0.005, a/d 0.002
Ethnicity, *M* (range)	1 (1)	1 (1)	1 (1)	1 (0)	a/c 0.011, a/d 0.001, b/c <0.001, b/d <0.001
LITE 90th percentile (c), *M* (range)	0 (1)	0 (1)	0 (1)	0 (1)	a/b <0.015, a/c <0.001, a/d 0.049

Four adaption groups were created by combining the lowest versus highest tertiles of risk scores and internalizing problems. Child internalizing problems were assessed at age 12 and proposed resource variables measured when the child was age 3

Dependent variables: CBCL (internalizing and externalizing symptoms). Independent variables: maternal sense of coherence, child social functioning, child temperament. Categorical variables: *5*-HTTLPR (0 = l/l, 1 = s-carriers), sex (1 = girls, 2 = boys) and ethnicity (0 = one or both parents born abroad, 1 = both parents born in Sweden), LITE (0 < 90th percentile, 1 ≥ 90th percentile)

*CELA* cumulative early life adversity, *SOC* sense of coherence, *5*-HTTLPR serotonin transporter gene-linked polymorphic region, *LITE* life incidence of traumatic events, *m* maternal variable, *c* child variable

**Table 4:** Model III: MANCOVA results—resource variable means or medians for the four adaption groups and planned contrast for externalizing problems

Resource variable	A. Resilient (high CELA + low ext probl) n = 77 M(SD)	B. Maladaptive (high CELA + high ext probl) n = 131 M(SD)	C. Competent (low CELA + low ext probl) n = 150 M(SD)	D. Highly vuln (low CELA + high ext probl) n = 74 M(SD)	Planned contrast a/b, a/c, a/d, b/c, b/d, c/d
SOC (m)	66.05 (10.90)	63.19 (11.82)	74.91 (8.77)	72.12 (9.11)	a/c <0.001, a/d 0.002, b/c <0.001, b/d <0.001
Social functioning (c)	17.61 (2.82)	15.93 (2.62)	17.85 (1.59)	17.20 (1.77)	a/b <0.001, b/c <0.001, b/d <0.001, c/d 0.027
Temperament (c)	23.00 (5.06)	20.00 (5.12)	25.02 (5.13)	22.69 (4.52)	a/b <0.001, a/c 0.012, b/c <0.001, b/d 0.001, c/d 0.004
*5*-HTTLPR (c), *M* (range)	1 (1)	1 (1)	1 (1)	1 (1)	NS
Sex, *M* (range)	2 (1)	2 (1)	1 (1)	2 (1)	a/c 0.001, b/c 0.003, c/d 0.029
Ethnicity, *M* (range)	1 (1)	1 (1)	1 (1)	1 (1)	a/c 0.001, a/d/0.022, b/c <0.001, b/d 0.012
LITE 90th percentile (c), *M* (range)	0 (1)	0 (1)	0 (1)	0 (1)	b/c 0.002

Four adaption groups were created by combining the lowest versus highest tertiles of risk scores and externalizing problems. Child externalizing problems were assessed at age 12 and proposed resource variables measured when the child was age 3

Dependent variables: CBCL (internalizing and externalizing symptoms). Independent variables: maternal sense of coherence, child social functioning, child temperament. Categorical variables: *5*-HTTLPR (0 = l/l, 1 = s-carriers), sex (1 = girls, 2 = boys) and ethnicity (0 = one or both parents born abroad, 1 = both parents born in Sweden), LITE (0 < 90th percentile, 1 ≥ 90th percentile)

*CELA* cumulative early life adversity, *SOC* sense of coherence, *5*-HTTLPR serotonin transporter gene-linked polymorphic region, *LITE* life incidence of traumatic events, *m* maternal variable, *c* child variable

Overall, no dramatic differences were detected regarding either internalizing or externalizing problems. As expected, the group which comprised of the competent children had a higher score on most proposed resilience variables compared to the other groups while the maladaptive group had the lowest score on most variables. Regarding internalizing problems, mothers of competent children had the highest scores on maternal sense of coherence compared to all other groups. Maladaptive children had the lowest scores of social functioning compared to the other groups, but apart from that differences were small. Competent children had the easiest temperament followed by resilient, highly vulnerable and maladaptive children, although the difference between resilient and highly vulnerable children was not significant. Highly vulnerable children were significantly more likely to be carriers of the s-allele of the *5*-HTTLPR compared to resilient and competent children (Table 3). There were more second generation immigrants in the resilient and maladaptive groups compared to the competent and highly vulnerable groups. There were significantly more boys in the resilient group than in the other groups. Resilient children had experienced traumatic life events to a lesser degree than all the other groups (Table 3).

Regarding externalizing problems, competent and highly vulnerable children had higher scores on maternal sense of coherence than resilient and maladaptive children. Maladaptive children had significantly lower social functioning scores compared to all other groups. Competent children had a higher score on social functioning compared to highly vulnerable children. Competent children also had the easiest temperament followed by resilient, highly vulnerable and maladaptive children, although the difference between resilient and highly vulnerable children was not significant (Table 4). There were more second generation immigrants in the resilient and maladaptive groups compared to the competent and highly vulnerable groups. There were significantly more girls in the competent group than in the other groups. Competent children had experienced traumatic life events to a lesser extent than maladaptive children (Table 4).

## Discussion

The present study applied a biopsychosocial model of risk and resilience to examine proposed resilience factors at preschool age and their impact on child behavior at age 12. Based on previous findings [36], we used a composite measure of risk adding maternal mental health problems, psychosocial risk and experience of multiple life events. As presumed, cumulative early life adversity increased the risk for both internalizing and externalizing problems. Also, bivariate analysis confirmed that most proposed resilience variables decreased the risk of behavioral problems, with the exception of *MAOA, COMT* and *BDNF* genotypes. The results of the study can be summarized in the following three main findings.

First, the l/l genotype of the *5*-HTTLPR was shown to decrease the likelihood for internalizing problems in the whole group. Interestingly this result persisted for the group with the lowest risk score, but not for the high risk group. Correspondingly, no interaction effect between cumulative adversities and *5*-HTTLPR was seen. When the four adaption groups were created, the resilient children had the most l/l carriers followed by competent children and the s-allele was most frequent among highly vulnerable children. These results indicate that the l-allele is somehow associated with lower emotional problem scores, but that the association is not mainly related to the level of risk. There are studies indicating a main effect of the *5*-HTTLPR on depression [52], however the focus has rather been on finding gene-by environment effects. Moreover, many previous studies used a more specific measure of adversity (e.g. childhood maltreatment) [28], whereas the cumulative early life adversity index used in the present study is a composition of several risk factors including maternal depressive symptoms and experience of life events and reflects a general stress load during early childhood. However, the main effect of *5*-HTTLPR on internalizing problems shown in the present study was consistent over the models, and the person-centered analysis revealed different allele distribution between the groups. Moreover, an effect of *5*-HTTLPR on externalizing problems was noted in the interaction model and in the residual model for the whole group, however no differences were found in the person centered model.

Secondly, among the other proposed resilience variables, an easy temperament at age 3 was shown to be associated with resilient outcomes at age 12, although the effect was not strong. Temperament was the only factor that decreased the risk for behavioral problems in the high risk group, but not the low risk group. This result is in line with previous results that state temperament as robustly associated with resilience [14, 17]. An easy temperament was the only factor specifically associated with resilient outcomes in the present study, whereas the other factors were beneficial for all children, or children with a low degree of adversity. For example, good social functioning was found to be more of a general resource factor, promotive for children with both low and high risk for behavioral problems. When comparing the four adaption groups, the only group that stood out regarding social functioning was the maladaptive group who had significantly lower scores compared to the others.

A high maternal sense of coherence decreased the risk for behavioral problems only in the total sample, however the p-value was approaching significance (*p* = 0.058) for the low-risk group for externalizing problems. When comparing the four adaption groups, mothers of competent and highly vulnerable children (both groups which had a low adversity index) had significantly higher SOC scores than resilient and maladaptive children (who had a high adversity index). Hence a low degree of early life adversity was associated with a high level of maternal SOC and vice versa. This could represent a direct association or a reciprocal relationship between cumulative life adversities and maternal SOC. Previous studies indicate that experiencing negative life events can alter SOC levels [53]. Effect sizes for maternal SOC were very small across all analyses, and a more recent measure of maternal SOC would have been valuable to further evaluate the impact of this factor.

Thirdly, as expected, girls had an increased risk for internalizing problems compared to boys, whereas boys had an increased risk for externalizing problems. Concerning internalizing problems, there were significantly more boys in the resilient group than in the other groups. For externalizing problems, more girls were found in the competent group compared to the maladaptive and highly vulnerable groups. No consistent pattern was seen for ethnicity across the models, however the person-centered model indicated that there were more second generation immigrants in the resilient and maladaptive groups compared to the competent and highly vulnerable groups. This could point toward an association between ethnicity and a high degree of early life adversity. The models were also controlled for experience of traumatic life events by age 12. Children who reported many traumatic life events had an increased risk for externalizing problems, while no consistent effect was seen for internalizing problems.

In summary, the present study found support for the importance of both biological and psychosocial factors measured at preschool age for resilient outcomes on mental health at the age of 12. However, effect sizes were small and none of the models explained much of the variance, indicating that child mental health/behavior is complex and that other factors contribute to the development of behavioral patterns. Furthermore, the effect of *5*-HTTLPR was not mainly related to the level of risk. Despite this, the present study indicates that resource factors present at preschool age can be of importance during preadolescence, and shows how different resource factors play different roles depending of the level of risk. The results are strengthened by the use of multiple methodologies which generally show similar results for most variables. Moreover, both individual and family factors were found to be associated with resilient outcomes in the context of child mental health. The present study did not include measures of the social environment; however, at the age of 3 the social environment is mainly limited to that of the family. At the end of the 90 s, 80 % of 3-year olds were enrolled in preschools in Sweden [54], and an assessment of the preschool quality and pro-social adult relationships would have added valuable information. To better understand the family environment at this age, an evaluation of mother–child attachment would have been interesting.

There are some limitations in the context of the present study that needs to be discussed. Unfortunately the present study was burdened with a dropout rate of 47.2 %. The dropout analysis revealed differences between participants and non-participants regarding ethnicity and experience of early life adversity. A high attrition rate is not uncommon in longitudinal studies [55], however since the dropout rate is somewhat skewed this can affect the analyses as the groups are divided based on the level of risk.

As mentioned above, effects were generally small, and none of the models explained a large variance (data not shown), indicating that the proposed variables only account for a small part of the pattern of behavior and resilience in 12-year old children. The results were expected to that extent that behavior is multifactorial, and that many other factors contribute to the wellbeing of 12-year olds. Some of the proposed resilience variables, i.e. sense of coherence and to some extent temperament, showed effect sizes that were statistically significant, however most likely of weak clinical significance. Consequently, the present study does not fully establish the relationship between all proposed variables and mental health in preadolescence, but for some variables rather indicates an association.

Moreover, a long period of time (9 years) elapsed between the measures for risk, resilience and outcome. Resilience is a dynamic concept, and a measure of mental health/behavioral problems at an earlier stage could have rendered other results. However the risk factors used have previously shown long-term effects on behavior [9, 10] and proposed resilience factors such as temperament have shown a relatively good stability over time [56]. Moreover, the biological measures used in the present study are constant; however, a future area of investigation could be epigenetic changes and resilient outcomes, as suggested by several researchers [57, 58]. The timespan also increases the risk for other factors to contribute to the pattern. For example, maternal depression after the postpartum period was not assessed, and could increase the risk for child behavioral problems. Despite this, the present study found significant associations between many of the proposed resilient factors and internalizing and externalizing problems in 12-year old children, suggesting that these factors still appear to be of importance for child development and mental health. The results can also be used to understand the impact of risk and resilience specifically at pre-school age, and the long-term consequences of the investigated factors.

The instruments were completed solely by the mothers (with the exception of the LSS which was assessed by a psychologist after interviewing the mothers) and no objective measures were used. There is a risk that the mothers own functioning affects both the way child behavior is reported and child behavior per se. However, in a previous study, maternal mental health was not shown to bias the results to a serious degree [59]. Moreover, at the age of 3 (and in most cases also at the age of 12) the parents would most likely be the ones who know their children the best. However, differences in reports on child behavior between mothers and fathers, as well as between parents and teachers have been shown earlier, and might be explained in part by that different behaviors become evident in different environments. Using parents’ reports instead of self-reports for assessing behavioral problems at age 12 could possibly increase the risk of underestimating internalizing problems which are not usually as evident to others such as parents and teachers, as are externalizing problems.

### Summary

In the present study, multiple statistical methodologies were used to examine a biopsychosocial model of risk and resilience on behavior at preadolescence. 889 children and their mothers were followed from birth to age 12. In summary, the results indicate the importance of both biological and psychosocial factors on resilient outcomes on mental health in preadolescence. It illuminates different patterns of interplay between resource factors and adversity in preadolescence. Moreover, the results supports the influence of *5-*HTTLPR on internalizing symptoms, however, not mainly related to the level of risk.
